# Does training with amplitude modulated tones affect tone-vocoded speech perception?

**DOI:** 10.1371/journal.pone.0226288

**Published:** 2019-12-27

**Authors:** Aina Casaponsa, Ediz Sohoglu, David R. Moore, Christian Füllgrabe, Katharine Molloy, Sygal Amitay

**Affiliations:** 1 Medical Research Council Institute of Hearing Research, Nottingham, England, United Kingdom; 2 Department of Linguistics and English Language, Lancaster University, Lancaster, England, United Kingdom; Universidad de Salamanca, SPAIN

## Abstract

Temporal-envelope cues are essential for successful speech perception. We asked here whether training on stimuli containing temporal-envelope cues without speech content can improve the perception of spectrally-degraded (vocoded) speech in which the temporal-envelope (but not the temporal fine structure) is mainly preserved. Two groups of listeners were trained on different amplitude-modulation (AM) based tasks, either AM detection or AM-rate discrimination (21 blocks of 60 trials during two days, 1260 trials; frequency range: 4Hz, 8Hz, and 16Hz), while an additional control group did not undertake any training. Consonant identification in vocoded vowel-consonant-vowel stimuli was tested before and after training on the AM tasks (or at an equivalent time interval for the control group). Following training, only the trained groups showed a significant improvement in the perception of vocoded speech, but the improvement did not significantly differ from that observed for controls. Thus, we do not find convincing evidence that this amount of training with temporal-envelope cues without speech content provide significant benefit for vocoded speech intelligibility. Alternative training regimens using vocoded speech along the linguistic hierarchy should be explored.

## Introduction

Temporal-envelope information is crucial for speech perception [1–10]. For instance, temporal-envelope (TE) information but not temporal fine structure (TFS) is preserved when hearing is restored through cochlear implants, and listeners can learn to use the impoverished signal produced by the cochlear-implant processor to extract speech information. The degree to which listeners are able to process the TE cues available in speech plays an important role in determining speech-perception outcomes. Cochlear-implant patients who are better at detecting TE modulations tend to be better at speech perception in quiet [11, 12], and specifically, at phoneme identification [13–16]. Likewise, Erb et al. [17, 18] found that listeners who were better at discriminating amplitude-modulation (AM) rates were quicker to improve in their perception of vocoded speech (see also [19]), which is often used to simulate the signal as heard through a cochlear implant. In vocoded speech, temporal envelopes are extracted from broad frequency regions, and used to modulate tone or band-limited noise carriers. This manipulation removes the original TFS whilst preserving lower-frequency TE modulations. Thus, learning of vocoded speech has important implications for a range of contexts where the auditory signal is degraded, such as in the case of hearing impairment.

Intelligibility of vocoded speech for listeners with normal hearing depends on several factors, but most importantly the number of frequency bands used for vocoding [20, 21]. When many frequency bands are used, intelligibility of noise- and tone-vocoded speech remains high, but exponentially degrades as the number of channels decreases [6, 7, 21, 22]. However, although vocoded speech represented by few channels (i.e., ≤ 6) is initially unintelligible for naïve listeners, it can become intelligible with training or repeated exposure [7, 23]. Perceptual learning of vocoded speech has also been shown to generalize to untrained sentences, words and nonwords [23–25]. For instance, Hervais-Adelman and colleagues trained participants in noise-vocoded word recognition [24] and sentence recognition [25], and showed generalization of learning to untrained words and nonwords [24] and to sentences in an untrained frequency region [25]. More recently, McGettigan et al [22] demonstrated a significant predictive role for low level acoustic-phonetic features in recognizing vocoded speech across the linguistic hierarchy, from phonemes to sentences. Listeners’ performance on a noise-vocoded consonant recognition task (consonants embedded in vowel-consonant-vowel contexts) was the best tested predictor of performance in other vocoded linguistic tasks (e.g., word and sentence recognition). These findings are in line with the results obtained using tone- and noise-vocoded speech stimuli by Loebach et al. [26–28], who postulated that training in low-level acoustic-phonetic listening offers the most promising route for adaptation to distorted speech. Thus, even though training on different linguistic levels leads to improved vocoded-speech perception, training on bottom-up processes related to TE cues available in vocoded speech seems a good candidate to promote transfer of learning to higher-level linguistic representations.

Perceptual learning of TE information without speech content (e.g., tones) has been observed after only two days of training [29]. Generalization to untrained stimuli has already been observed across different modulation rates and, to some extent, across carriers (see [29–32]). However, generalization across tasks with similar stimulus features (e.g., discrimination of a change in rate versus detection of a modulated carrier) has been observed only for some tasks (see [29, 30]). Similarly, vocoded speech recognition learning has been shown to generalize across different frequency regions but only across some carrier types [25]. Learning and generalization of TE perception with and without speech content has been observed after brief periods of training and several studies have shown a tight relationship between participants’ ability to detect TE information and speech perception (see [11–19]). However, the generalization of training to higher linguistic levels using TE cues in low-level tasks has not previously been studied.

One of the pioneer investigations exploring the relationship between training on low-level tasks and performance on different language outcomes was the study by Merzenich and colleagues [33]. They showed that children with language-based learning impairment (LLI) improved in their ability to recognize phonetic elements within words after training on temporal processing information of speech and non-speech materials. They also showed that the intensity of training required was directly related to participants’ performance on language outcomes (Token Test for Children, Teaching Resources Corporation, Boston, MA, 1978), suggesting a link between perceptual temporal processing and language comprehension.

Given the importance of TE cues to both aided and unaided speech perception, we asked here whether (1) listeners’ ability to perceive these envelope cues in non-speech stimuli is related to initial learning of vocoded speech, and (2) training on these cues can transfer to improved perception of spectrally degraded speech. To investigate this, we trained listeners on either detection of TE modulation (sinusoidal AM) or the discrimination of the AM rates, and tested for improvement in perception of vocoded vowel-consonant-vowel (VCV) ‘nonsense’ syllables. These sub-lexical stimuli were chosen to minimize the influence of higher-level linguistic structure (e.g., semantic or syntactic) on learning effects. We predicted that (1) listeners’ performance on AM tasks prior to training will be correlated with their ability to quickly adapt to vocoded VCV segments on initial exposure (pre-training), and (2) training on TE cues without speech content would improve vocoded consonant identification when compared to untrained, control listeners.

## Methods

### Ethics statement

The research protocol was approved by the Nottingham University Hospitals NHS Trust Research Ethics Committee. Written informed consent was obtained from all participants prior to the first experimental session, and they were paid an inconvenience allowance for their participation.

### Participants

Fifty-four native English speakers (27 females) aged 18–40 years (mean = 22.9, standard deviation, SD = 5.9) were recruited via posters from the University of Nottingham student population and the general public. No participants reported any history of language disorders or hearing impairment. Screening confirmed that bilateral audiometric pure-tone thresholds were ≤ 20 dB Hearing Level at octave frequencies across 0.25–4 kHz in both ears. No participants had prior experience of psychoacoustic testing or vocoded speech.

### Equipment

Testing took place within a double-walled sound-attenuating booth. Tasks were based on custom software written in MATLAB Release R2007a (The MathWorks Inc., Natick, Massachusetts). Stimuli were presented diotically at 70 dB sound pressure level (SPL) using Sennheiser HD-25-I headphones. Participants responded to vocoded speech recognition tasks via a touchscreen LCD computer monitor placed in front of them at a comfortable distance. Responses to psychophysical tasks were recorded via a custom-made button box placed horizontally in front of the listeners.

### General protocol

**Fig 1:**
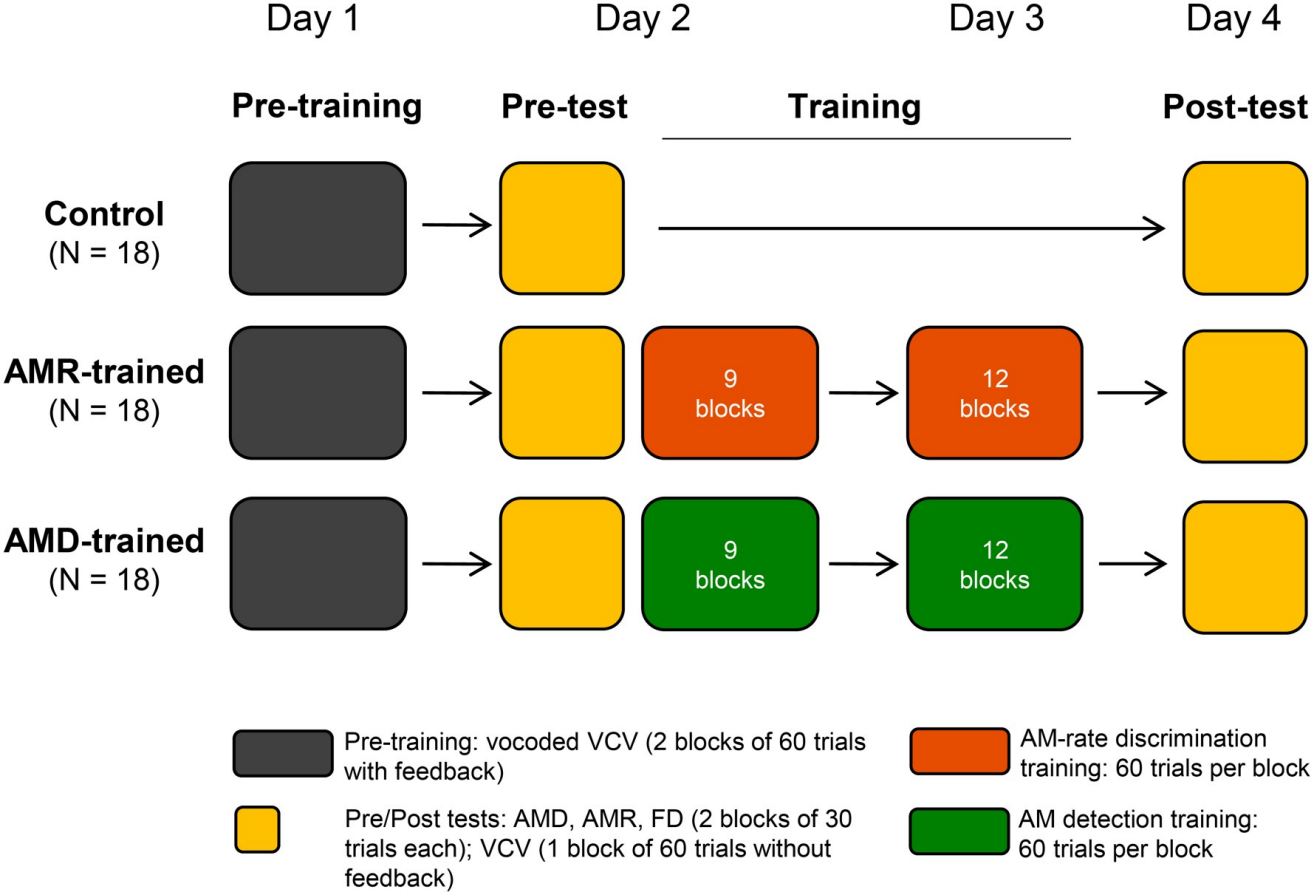
Schematic representation of the different regimens.

The study was conducted over four consecutive days and the protocol consisted of a pre-training phase, a pre-test, a training phase, and a post-test (Fig 1). During pre-training, all participants performed two blocks of a tone-vocoded speech recognition task (see below) with feedback. This pre-training phase was included, as previous work has shown that rapid learning occurs when listeners are first exposed to vocoded speech [19, 23, 24], which could mask experimental effects arising from AM-based training. We anticipated that after the learning occurring in this pre-training phase, listeners’ speech identification scores would plateau to a more stable level. The resulting performance scores were used to assign each listener to one of the three experimental groups (N = 18 each) ensuring balanced initial vocoded-speech recognition performances across groups. All groups took part in the pre- and post-tests during which they were tested on vocoded consonant identification, AM-detection (AMD), AM-rate discrimination (AMRD), and frequency discrimination (FD) tasks. The latter task was included to test the degree of specificity of AM-specific learning. Between pre-test and post-test, one group completed two training sessions on an AMD task (‘AMD-trained’) and a second group trained on the AMRD task (‘AMR-trained’), administered on two consecutive days. The remaining group acted as untrained controls and completed only the pre-test and post-test.

Since previous studies have shown a relationship between listeners’ memory span and performance on AM tasks, as well as the perception of vocoded speech (e.g., [17, 34]), all participants were also administered two measures of memory span (i.e., the Forward Digit Span and Backward Digit Span subtests of the Wechsler Abbreviated Scales of Intelligence [35]).

### Pre-training phase

#### Vocoded consonant identification (with feedback)

All participants undertook pre-training on the vocoded consonant identification task. This phase allowed us to match the groups on initial average speech identification performance by assigning participants to each group according to their baseline performance and thus avoid bias due to poorer listeners showing greater learning (as has been previously found: [36]; see also [37–39]). This was achieved by calculating individual percentage of correct responses after the completion of the second block of tone-vocoded VCV stimuli (i.e., pre-training2). First, each participant was initially pre-assigned to one of the groups in a counterbalance manner. Then, we calculated participant’s percentage of accuracy on pre-training2 vocoded consonant identification task. If the mean of the current participant was considerably lower than the individual means on each of the three groups, then the participant was re-assigned to the group with overall higher mean. Similarly, if the mean of the participant was considerably higher than the individual means on each of the groups, then the participant was re-assigned to the group with lower mean (see S1 Table for mean group percentage of accuracy in Pre-training2 alongside SD and 95% confidence intervals). If the mean of the participant was within the mean range of the groups, participant was kept in the pre-assigned group.

Participants completed two blocks of 60 tone-vocoded VCV stimuli separated by a 10-min break. In each trial listeners were asked to identify the consonant in a 20-alternative, forced-choice procedure. The 20 possible consonants (see below) were represented orthographically on a touch-screen and participants were instructed to select the response alternative corresponding to the consonant they heard. There was no time limit for entering the responses and participants were encouraged to guess if they could not identify the consonant. After each response, auditory and visual feedback were given in the form of the clear unprocessed version of the stimulus, followed by a repeat presentation of the vocoded VCV and the correct consonant highlighted in green on the screen. The presentation of the stimuli was fully randomized for each participant and block. To familiarize participants with the task, five practice trials were presented before the first block.

Each of the 20 consonants (/b/, /ʧ/, /d/, /f/, /g/, /ʤ/, /k/, /l/, /m/, /n/, /p/, /r/, /s/, /ʃ/, /t/, /θ/, /v/, /w/, /j/ and /z/) was embedded in three vowel contexts (/a/, /i/ and /u/). The resulting 60 VCV stimuli were spoken by a male native British-English speaker and recorded on a digital audio tape recorder. The recordings were digitalized with 16-bit resolution at a sampling rate of 22.05 kHz. Each VCV stimulus was vocoded using Tiger CIS software written by Qian-Jie Fu (www.tigerspeech.com, Version 1.05.02). Speech stimuli were divided into six contiguous frequency bands equally spaced on the ERB_N_-number scale. Corner frequencies (3-dB down from the peak of the passband) of the filters were at 100, 331, 725, 1396, 2538, 4485 and 7800 Hz with a roll-off of 24 dB per octave. The frequencies of the 6 channels were centred at 185, 491, 1007, 1881, 3375, and 5916 Hz. The slow-varying envelope within each band was extracted by removing pitch-synchronous oscillations above 45 Hz (with a roll-off of 24 dB per octave) and used to modulate sine waves located at the logarithmic center frequency of each analysis band. Although classic psychophysical studies [3] suggest that AM rates up to 16 Hz are the most important for speech intelligibility, information related to some phonetic features of consonants can go up to 50 Hz (e.g., place of articulation), hence we included here modulation rates up to 45 Hz. Pure-tone carriers were used instead of noise carrier, because the latter contain extraneous envelope fluctuations that might interfere with amplitude modulation applied to the signal [21, 40]. The modulated sine waves were combined to produce the final vocoded VCV stimuli. See S1–S6 Audio Files for examples of clear and vocoded VCV stimuli.

### Pre- and post-tests

#### Vocoded consonant identification (without feedback)

The same task undertaken during the pre-training was used for the pre- and post-tests, except that no feedback was provided to minimize further learning during the tests. Participants performed one block (i.e., 60 VCVs) during the pre-test and the post-test.

#### AM detection (AMD)

Listeners performed two blocks of 30 trials employing a three-interval three-alternative forced-choice (3I-3AFC) oddball design with feedback. Two standard intervals contained an unmodulated carrier, and the third, randomly determined interval (the target), contained an AM carrier. Listeners were instructed to detect the interval that was different from the other two (see [29] for similar procedure).

AM stimuli were created by multiplying a 1-kHz pure-tone with DC-shifted sinusoids of the required modulation rate. For the pre- and post-tests, a modulation rate of 8 Hz was used to maximize the potential transfer of learning to vocoded consonant identification, as this corresponds to temporal information at the sub-syllabic level (average syllabic rate in English = ~5 Hz; [3, 41–44]). The duration of each tone was 500 ms, including 10-ms raised cosine ramps, with an inter-stimulus interval of 750 ms. The amplitude of all AM tones was reduced by [1 + m^2^/2]^0.5^ (where *m* is the modulation depth) to cancel out the increase in power resulting from amplitude modulation, and thus avoid participants’ decisions based on intensity cues [45]. The starting phase of modulation was randomized in an interval-by-interval manner.

In the AMD task the modulation depth (*m*) was initially set to 100% (0 dB), then adaptively adjusted by a factor of 1.58 (4 dB) according to a one-down, one-up staircase procedure. After the first incorrect response, the factor was changed to 1.26 (2 dB) and an adaptive three-down, one-up staircase procedure, targeting 79% correct on the psychometric function [46] was used until the total number of trials have elapsed. Before the first block, participants underwent practice of five trials with three trials being ‘easy’ (*m* = 100%), and two ‘impossible’ (*m* = 0%).

#### AM-rate discrimination (AMRD)

The procedure and task for AMRD was similar to those used for AMD. Two blocks of 30 trials were presented to each listener employing a 3I-3AFC oddball design with feedback. The two standard intervals consisted of 1-kHz carriers modulated at 8 Hz (*m* = 100%) and the target interval contained the same carrier modulated at a higher rate. The modulation rate (*f*_*m*_) for the target varied adaptively (8 Hz + Δ*f*_*m*_), starting at Δ*f*_*m*_ = 50% of the standard rate (i.e., 4 Hz) and was reduced by a factor of 1.4, according to a one-down, one-up staircase procedure. After the first incorrect response, the step size was changed to √1.4 and a three-down, one-up staircase procedure. Five practice trials were presented before the first block to familiarize participants with the task. Three of these trials were ‘easy’ (Δ*f*_*m*_ = 50%) and two were ‘impossible’ (Δ*f*_*m*_ = 0%).

#### Frequency discrimination (FD)

Two blocks of 30 trials were administered to every listener employing the same 3I-3AFC oddball design with feedback. The frequency of the two standard tones was 800 Hz while the frequency of the target, randomly determined interval contained a higher-frequency (800 Hz +Δ*f*). Listeners were instructed to indicate the interval that was different in pitch. Starting with Δ*f* = 50% (i.e., 400 Hz), Δ*f* of the subsequent trial was divided by two until the first incorrect response. Thereafter, the step size was √2. Before the first block a five-trial demonstration was administered. Three of these were ‘easy’ (Δ*f* = 50%) and two impossible (Δ*f* = 0%). FD stimuli consisted of 100-ms tones (including 10-ms raised cosine ramps) with an inter-stimulus interval of 500 ms. This task was chosen as a control psychophysiological task in order to show the degree of specificity of the AM training regimes, and to be able to measure any disruption of learning due to training on AMD or AMRD tasks.

### Training phase

One group was trained on an AMD task (‘AMD-trained’ group) and a second group was trained an AMRD task (‘AMR-trained’ group), using similar adaptive procedures to those used for the test phases. Participants completed 21 blocks of 60 trials spread across two days. Nine blocks were completed on Day 2 (immediately following the pre-test) and an additional 12 blocks were completed on Day 3. For both AM training tasks, the standard *f*_*m*_ varied pseudo-randomly between 4, 8 and 16 Hz in a block-by-block manner using a Latin square design. These modulation frequencies were chosen because they are in the syllabic and sub-syllabic–the fluctuation rate at which the segments within a syllable (e.g., VC unit) occur–speech ranges that are critical for performing the speech perception task (vocoded consonant identification). In English the syllabic rate is around ~4 Hz [19, 41], the sub-syllabic rate can go up to ~16 Hz (e.g., sub-beat and consonants [43]), and the phoneme rate can go up to 50 Hz (see [9, 44]). The task used for training was the same than for pre- to post-tests (i.e., 3I-3AFC) as well as the staircase procedure, stimulus duration, and inter-stimulus interval.

### Statistical analysis

Analyses were conducted using the *lme4* [47] package in R [48]. Continuous outcome measures were analyzed with linear mixed-effect models (*lme*). Significance p-values and Type III F-statistics for main effects and interactions were calculated using Satterthwaite approximations to denominator degrees of freedom as implemented in the *lmerTest* package [49]. For the analysis of binary outcomes, mixed-effects logit models were employed [50]. Type III Wald χ^2^-statistics for main effects and interactions were calculated using the *car* package [51]. We also provide estimates (β), standard errors, and CI (95%) in addition to inferential statistics since binary decisions (significant, not-significant) with small samples sizes need to be considered cautiously. It is worth noting that due to the assumption of infinite number of degrees of freedom associated with mixed-effects logit models, we calculated confidence level (95%) for estimates resulting from binary dependent variables using the asymptotic method. This method, however, tends to make confidence intervals slightly too narrow as well as the p-values associated with it slightly lower.

All models included group (control, AMD-trained, AMR-trained) and test (pre-test, post-test) as fixed effects, including all main effects and their interactions. Additionally, information transfer analyses also included type of phonetic feature (voice, manner, place) as a fixed effect, including all main effects, 2-way and 3-way interactions. All models included maximal within-unit random effects structure [52, 53], thus random intercepts and random slopes for all the within-unit variables and interactions were included for participants, and items when suitable (see S1 Appendix for further model specifications). Control group and pre-test were used as reference levels for initial models. We also included control variables related to the task itself as fixed effects without the interaction terms (i.e., control variable block in psychophysiological tasks, and control continuous variable trial-order for vocoded consonant identification tasks; see S1 Appendix).

#### Psychophysical data

Raw threshold data from psychophysical measures (i.e., FD, AMD and AMRD) were log_10_-transformed and fitted with a psychometric function using the Psignifit toolbox [54]. Tracks where the optimization procedure did not adequately fit the data (i.e., negative slopes or fitted threshold values outside the measure range) were discarded (3.2% of the data; see S1 Dataset). This resulted in the exclusion of one participant from the AMRD data analysis due to lack of adequate fitted tracks for the post-test. Additionally, listeners’ thresholds that deviated more than 2.5 interquartile-range above and below the 3^rd^ and 1^st^ quartile from each group and task were considered outliers and excluded from further analysis (1.9%). On average the number of reversals per block of 30 trials (pre- to post-test phase) in each task was: AMD = 5.53 (SD = 1.72); AMRD = 5.11 (SD = 1.63); FD = 5.70 (SD = 2.16). Threshold estimates for the two consecutive blocks of 30 trials in pre- and post-tests were highly correlated: AMD pre-test: r = .75, p < .001, AMD post-test: r = .62, p < .001; AMRD pre-test: r = .70, p < .001, AMRD post-test: r = .53, p < .001; FD pre-test: r = .74, p < .001, FD post-test: r = .89, p < .001.

The threshold estimates for each task were analyzed using a group (Control, AMD-trained, and AMR-trained) × test (pre-test and post-test) mixed-effects model. Since all participants conducted two blocks of each task in the pre- and post-test sessions, this factor (i.e., block) was included as a control variable in the fixed-effect structure of the model [see 52].

#### Vocoded consonant identification

Mean percent correct score on the consonant identification task during the pre-training phase was used to assign each participant to one of the three experimental groups, ensuring balanced average initial identification performance for vocoded consonants (Control: 55.1%, SD = 49.8, confidence interval (CI; 95%) = 23.41–69.51; AMD-trained: 58.9%, SD = 49.2, CI (95%) = 22.0–70.9; AMR-trained: 63.3%, SD = 48.2, CI (95%) = 22.7–70.2; see S1 Table). There were no reliable statistical differences across groups in this task at this stage (χ^2^ (2) = 1.93, p = .38). It is worth noting that pre-test intercepts were not statistically different between trained groups and the control group (AMD-trained vs control: Estimate intercept difference = .30, SE = .32, z = .94, p = .35; AMR-trained vs control: Estimate intercept difference = .49, SE = .32, z = 1.52, p = .13 (see S1 Appendix—Table N), nor the descriptive statistics for pre-training scores showed noticeable differences given our sample size (Control: 56.0%, SD = 49.7, CI (95%) = 14.5–59.0; AMD-trained: 60.8%, SD = 48.8, CI (95%) = 13.1–60.4; AMR-trained: 63.0%, SD = 48.3, CI (95%) = 13.8–59.7 (see S1 Table).

For pre- to post-test analysis, listeners’ percent correct responses that deviated more than 2.5 interquartile range above the 3^rd^ and below the 1^st^ quartile of the group were considered outliers and discarded (3.7%). Due to an experimenter error one participant performed the consonant identification task with feedback in the post-test, and therefore was excluded from further analyses (see S2 Dataset for raw data). The remaining data were analyzed using a 3 (group) × 2 (test) mixed-effects model, checking for significant improvement for AM-trained groups compared to controls. We included trial order in the vocoded consonant identification task as a control predictor in the fixed structure of the model [see 52]. This allowed us to take into consideration the spontaneous learning on vocoded speech that occurs during the session.

#### Information transfer analyses (IT)

Information-transfer (IT) analysis [55] was conducted to investigate whether training on TE cues improved vocoded speech perception of phonetic features that are specially conveyed by TE information (i.e., manner and voice) [22, 56–58]. IT analysis provides the percentage of bits of information received by the listener. Through the use of confusions (such as /p/ being mistaken for /t/, where the type of voicing and manner have been correctly transmitted but not the place of articulation), we can estimate the extent to which different phonetic features are accurately transmitted to the listener. The categories for the phonetic feature of voice were voiced and voiceless. The categories for manner were nasals, fricatives, approximants and consonants starting with a plosive sound. For place of articulation three categories were selected: front, middle and back (see S2 Appendix). Each phonetic feature has a different probability of being correctly transmitted due to the different number of categories (e.g., voice has 50% of guessing correctly, whereas place of articulation has 33%) and the different probabilities within category (e.g., from 20 consonants eight were voiceless and 12 voiced), making it difficult to estimate what fraction of the input is correctly received by the listener. To overcome this problem, we computed the relative IT by dividing IT values obtained from individual confusion matrices by the input entropy (see S2 Appendix) [55, 59]. Relative IT percentages were then analyzed exploring the interaction between phonetic feature (voice, manner and place of articulation), group (Control, AMD-trained and AMR-trained), and test (pre- and post-test).

## Results and discussion

### Correlations between performance on AM tasks and vocoded consonant identification

Contrary to our prediction and the findings from Erb and colleagues [17, 18], pre-training improvement on vocoded-VCV identification was not significantly correlated with listeners’ initial performance on AMD (r = .039, t(50) = .28, p = .78) or AMRD (r = .004, t(51) = .03, p = .98). Our data suggested that early learning of vocoded VCVs does not seem to be reliably related to sensitivity to non-speech TE cues. It is noteworthy that differences in sample sizes between Erb et al. [17] and the current study might contribute to discrepancies in resulting correlations (18 participants in Erb et al. [17], 51 (AMD task) and 52 (AMRD task) in the current study). This discrepancy with previous work might also be due to the type of speech stimuli used in the two studies. Erb et al. [17] used sentence stimuli, which contain more linguistic information than our stimuli (e.g., lexical information or semantic predictability [22–24]). Furthermore, correct word identification in sentences might also rely on other cognitive processes (e.g., working memory [4, 24]) than in the current identification task (see S2 Table for correlations between vocoded speech and cognitive processing). Yet another factor that might contribute to divergent results between the studies could be the type of vocoder. Our study used tone-vocoded speech whilst noise-vocoded speech was used in Erb et al. Although previous studies had found that consonant recognition in quiet using VCV stimuli is not affected by the type of vocoder used [8, 40, 60], differences have been observed for words and sentences in quiet [40].

### Learning and transfer-of-learning on psychophysical tasks

**Fig 2:**
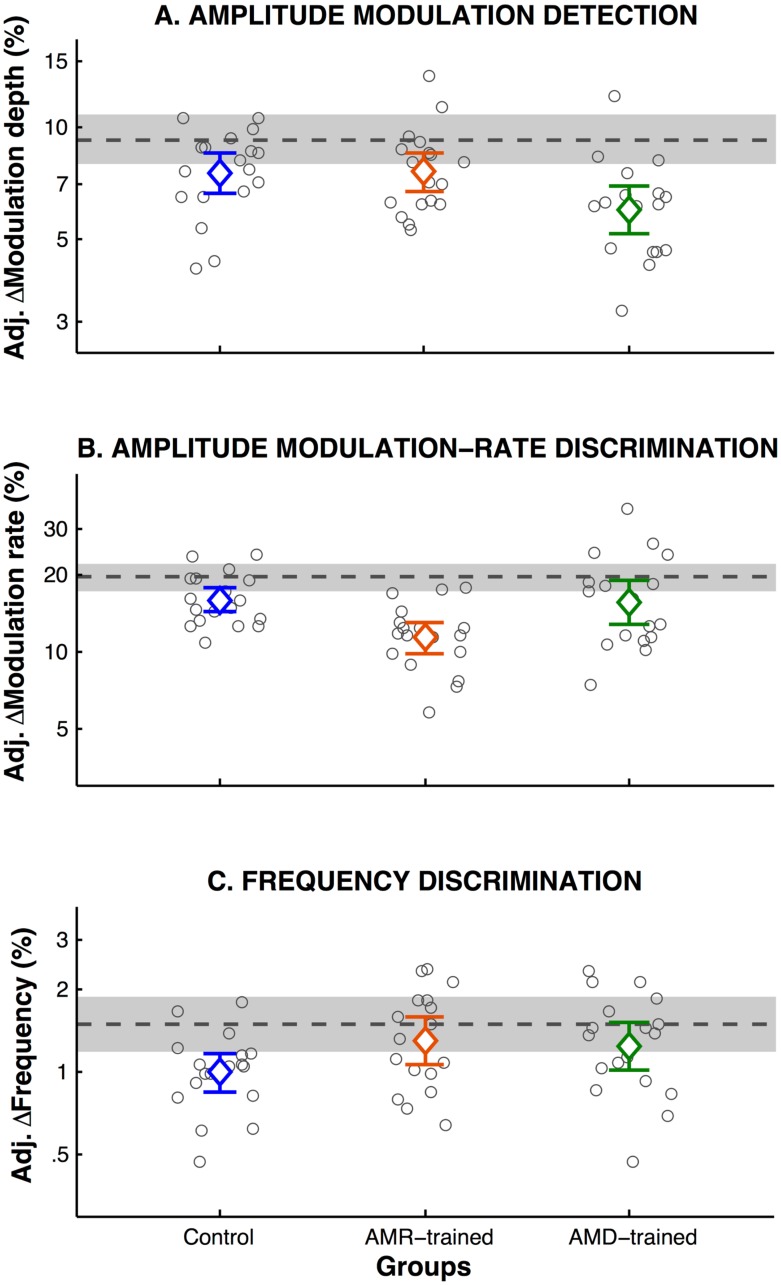
Learning and transfer of learning on psychophysical tasks. Mean adjusted post-test thresholds in percentage of improvement from pre- to post-tests at the individual (small circles) and group level (big circles) on (**A**) AMD, (**B**) AMRD and (**C**) FD tasks. Mean pre-test threshold across all participants is plotted as a dotted line, with the grey shaded area denoting the 95% confidence interval.

Prior to training the groups did not differ statistically on AMD (F(2,50.1) = .63, p = .53), AMRD (F(2,50.3 = .37, p = .69) or FD (F(2,51) = .69, p = .51), nor the summary-based statistics showed reliable differences across groups (see S1 Fig). Learning was observed in all three psychophysical tasks (Fig 2 and S1 Fig; main effect of test: AMD: F(1,50.2) = 23.3, p < .001; AMRD: F(1,50) = 41.91, p < .001; FD: F(1,103.5) = 15.76, p < .001). However, only in the trained tasks (AMD and AMRD) was there a modulation of learning by type of training (group × test interaction: AMD: F(2,50.2) = 3.18, p = .050; AMRD: F(2,50) = 4.21, p = .020; FD: F(2,103.5) = 1.52, p = .22).

Only the AMD-trained group showed reliable improvement on the AMD task (Fig 2A; AMD-trained: β = .21, SE = .05, CI (95%) = .12 –.31, t = 4.76, p < .001; AMR-trained: β = .07, SE = .04, CI (95%) = -.01 –.16, t = 1.69, p = .10; Control: β = .08, SE = .04, CI (95%) = -.01 –.17, t = 1.84, p = .08), and this improvement was significantly greater than both the control group (β = .13, SE = 0.06, CI (95%) = .01 –.26, t = 2.07, p = .04) and the AMR-trained group (β = .14, SE = .06, CI (95%) = .02 –.27, t = 2.25, p = .03), which did not show reliable differences (β = .01, SE = .06, CI (95%) = -.11 –.14, t = .20, p = .86; see S1 Appendix Tables A–E).

All groups showed significant improvement on the AMRD task (Fig 2B; AMD: β = .09, SE = .04, CI (95%) = .01 –.17, t = 2.34, p = .023; AMRD: β = .23, SE = .04, CI (95%) = .16 –.31, t = -6.12, p < .001; Control: β = .10, SE = .04, CI (95%) = .03 –.18, t = 2.77, p = .008). However, the AMR-trained group showed greater improvement than both the control group (β = .13, SE = .05, CI (95%) = .02 –.24, t = 2.32, p = .024) and the AMD-trained group (β = .16, SE = 0.06, CI (95%) = .05 –.27, t = 2.84, p = .007), which did not substantially differ (β = .03, SE = 0.06, CI (95%) = -.08 –.14, t = .57, p = .57).

Only the control group showed significant improvement on FD (Fig 2C; Control: β = .18, SE = .05, CI (95%) = -.08 –.27, t = 3.75, p < .001; AMD-trained: β = .08, SE = .05, CI (95%) = -.02 –.17, t = 1.62, p = .11; AMRD-trained: β = .07, SE = .05, CI (95%) = -.02 –.17, t = .17, p = .13), but this improvement did not reliably differ from the AM-trained groups, resulting in the non-significant group × test interaction (F(2,103.47) = 1.52, p = .22; Improvement differences: Control–AMD-trained: β = .11, SE = .06, CI (95%) = -.02 –.23, t = 1.65, p = .10; Control–AMRD-trained: β = .11, SE = .07, CI (95%) = -.02 –.24, t = 1.68, p = .10). It is worth noting that lower and upper confidence intervals for the difference of improvement in this task across AM-trained groups and controls show that the “true” population mean difference between the control group and AM-trained groups could be zero.

In summary, although there was some improvement on untrained tasks, only the AM-trained groups showed improvements that significantly exceeded that of the control group on the trained task, suggesting a degree of learning specific to the training task.

The observed learning for both AMD-trained and control groups from pre- to post-tests in the AMRD task, suggests that learning to discriminate AM rates can be captured with little exposure to the stimuli. Such rapid learning on the AMRD task could reflect faster perceptual and/or procedural learning (see [61]; see also [62]). Additional training on this task (AMR-trained group) also produced greater improvement compared to controls and the AMD-trained group, reflecting perceptual learning specific to stimulus processing and task, as also shown for the AMD-trained group on the AMD task.

Although the control group improved on the FD task, the improvement did not significantly exceed that of the AM- trained groups. Hence, these data do not support disruption of learning in FD tasks by AM training or of transfer of learning across tasks (see [32] for a review). These results are consistent with previous literature with similar samples sizes [61].

### Transfer of learning to vocoded consonant identification

**Fig 3:**
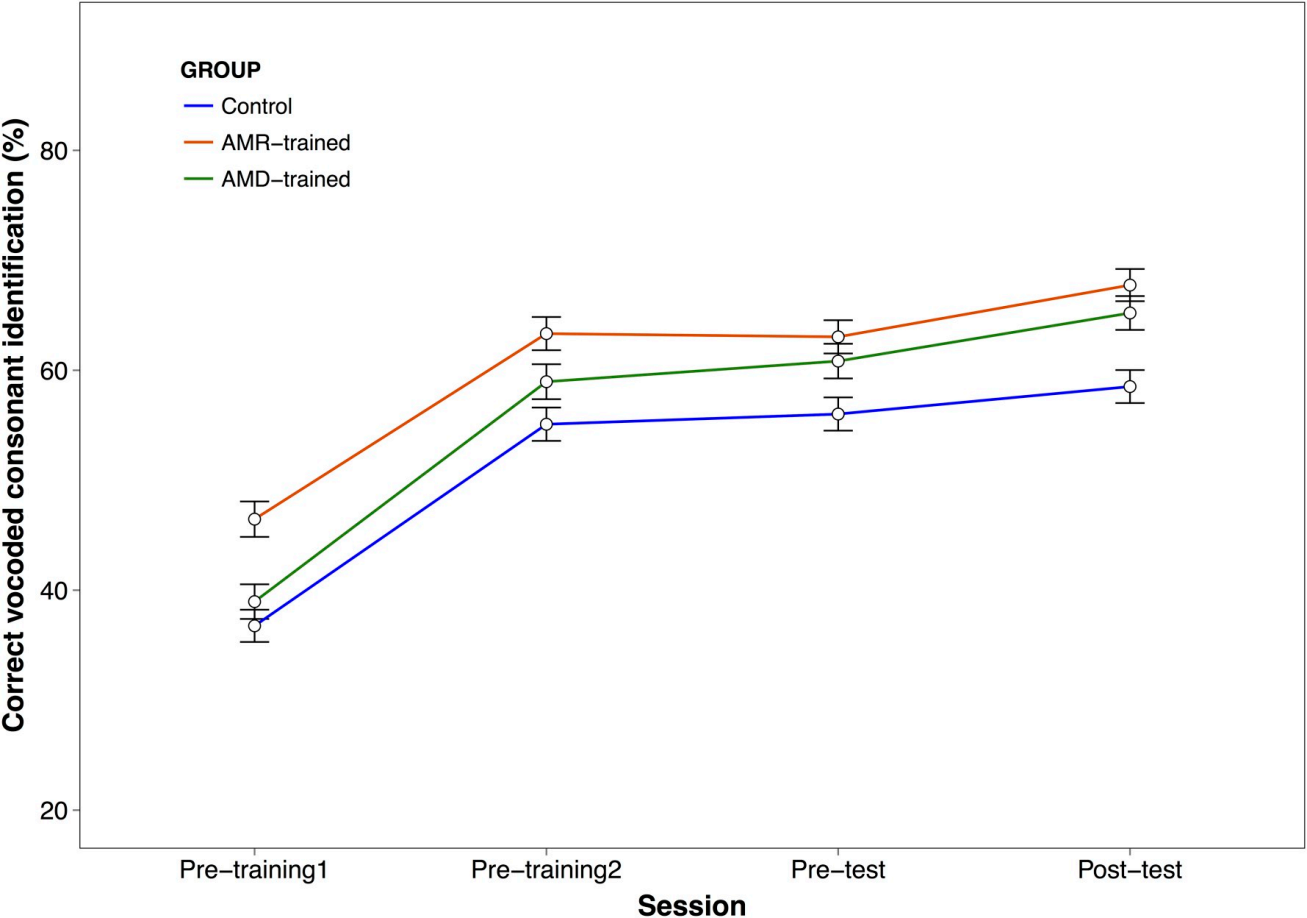
Percentage of correct vocoded consonant identification during pre-training and pre- to post-test. Learning curves for each group of participants across pre-training (two blocks), and pre- to post-tests of vocoded consonant identification task. Error bars represent +/- 1 standard error. In pre-training1 and 2 (Day 1) participants received online feedback during the task, but not in pre-test or post-test. Note that participants were split into the different groups after pre-training2 based on their mean scores ensuring balanced performance across groups before starting the training regimens. Learning from pre-training1 to pre- training2 was significant for all groups (all z values > 7, all p values < .001), whereas only AM-trained groups showed significant improvement from pre-test (Day2) to post-test (Day4).

AM-trained groups, but not controls, showed a significant improvement on vocoded consonant identification between pre- and post-test (Fig 3; AMD: β = .32, SE = .12, CI (95%) = 0.08–0.56, z = 2.55, p = .011; AMRD: β = .36, SE = .13, CI (95%) = 0.12–0.61, z = 2.87, p = .004; Control: β = .18, SE = .11, CI (95%) = 0.05–0.39, z = 1.54, p = .12). However, the improvement shown by the AM-trained groups was not reliably greater than that shown by the controls (group × test: χ^2^ (2) = 1.39, p = .50). It is worth noting the large and overlapping confidence intervals for the differences in improvement from pre- to post-test in each group, with lower confidence intervals close to 0 values even for AM-trained groups. As can be also seen in Table 1, group comparisons before and after training showed no reliable numerical differences between groups during pre-test and post-test sessions given our sample size and the variability within groups during all sessions (see S1 Table). In this regard, post-test mean estimates differences across groups show confidence intervals crossing the 0 value, suggesting that the mean population difference after training across groups could be zero. It is worth noting however, that learning kept occurring during performance of the VCV task itself (main effect of trial order: χ^2^ (1) = 12.5, p < .001; see S1 Appendix). Further analyses using Bayes Factors for the magnitude of improvement between groups after training (see [63]) show that the null hypothesis (i.e., no differences between groups) was around 2.5 times more likely than the alternative (Control vs AMD-trained: BF01 = 2.70; Control vs AMR-trained: BF01 = 2.61).

**Table 1:** Transfer of learning to vocoded consonant identification. Descriptive statistics including estimates (β), standard errors (SE), and asymptotic lower and upper confidence intervals, and inferential statistics for between groups comparisons during pre- and post-tests.

Test	Group	Estimate	SE	Lower CI	Upper CI	z-ratio	p-value
Pre-test	Control vs AMD-trained	-.30	.32	-1.04	.44	-.95	.61
	Control vs AMRD-trained	-.49	.32	-1.24	.26	-1.52	.28
	AMD-trained vs AMRD-trained	-.19	.33	-.96	.59	-.57	.83
Post-test	Control vs AMD-trained	-.44	.35	-1.27	.39	-1.25	.43
	Control vs AMRD-trained	-.68	.35	-1.50	.14	-1.93	.13
	AMD-trained vs AMRD-trained	-.24	.36	-1.08	.61	-.66	.79

#### Information transfer analysis

**Fig 4:**
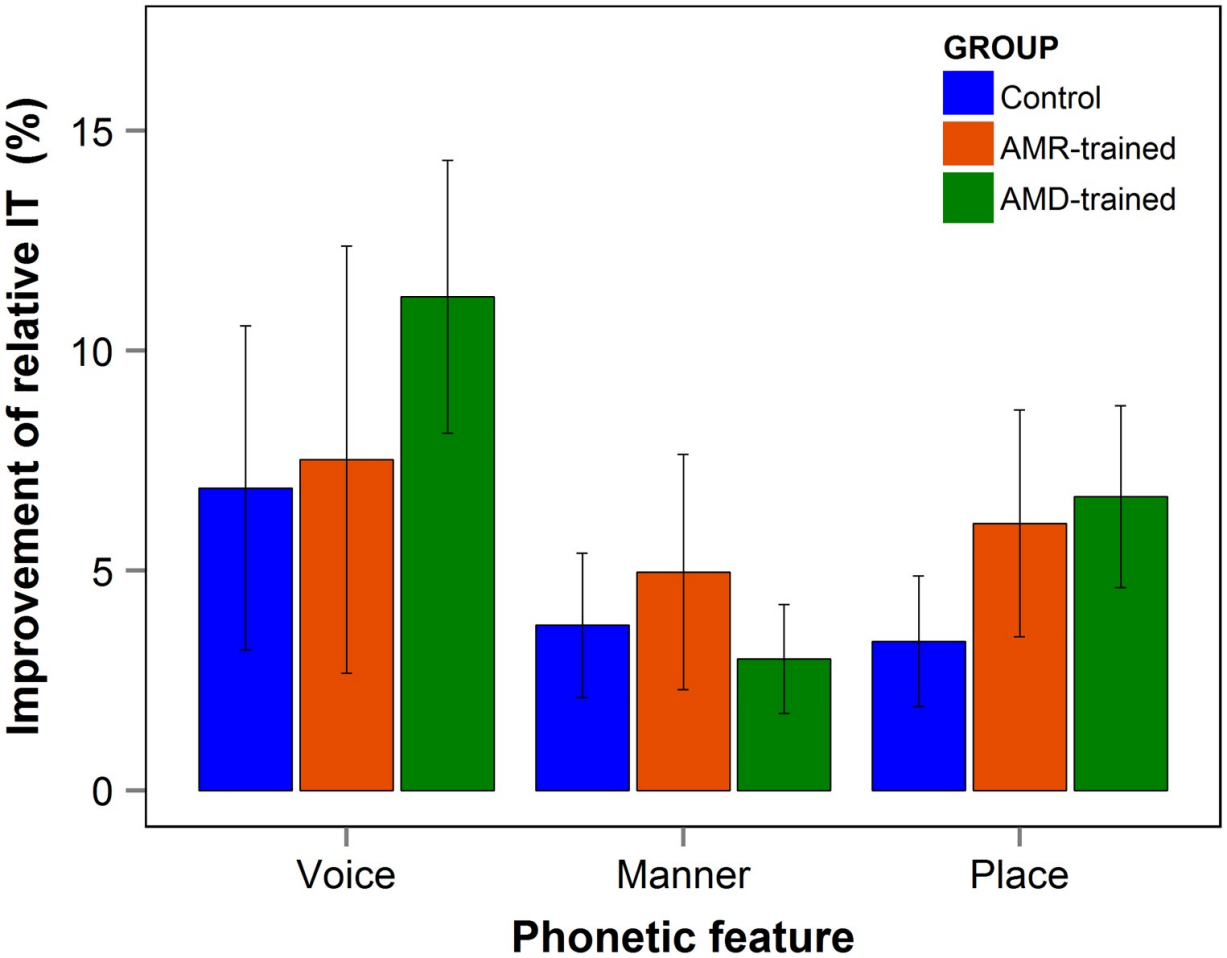
Relative information transfer. Improvement on relative information transfer for each phonetic feature and group. Error bars represent standard errors.

We next examined whether training improved specific phonetic features of the consonant identification task based on information transfer for voice, manner and place of articulation. We found a significant main effect of phonetic feature (F(2,240) = 492.6, p < .001), with manner best received (77.5%), followed by voice (73.9%) and place of articulation (32.5%), consistent with results reported by Lorenzi et al. (2000). Pre- to post-test learning was also significant (Fig 4; F(1,155.8) = 19.0, p < .001), showing an overall 5.9 percentage-point improvement (AMD-trained: 7.0%; AMR-trained: 6.2%; Control: 4.9%). However, neither the main effect for group (F(2,48) = .95, p = .39), nor the 3-way interaction between group, test and phonetic feature were significant (F(4,240) = .17, p = .95). These findings suggest that AM training did not improve specific phonetic features beyond those found in the Control group.

## General discussion

Given the importance of TE cues for both aided and unaided speech perception, and the results of previous studies, we expected to find an improvement on vocoded speech perception through explicit training on TE cues in non-speech sounds. However, despite robust learning on the trained AM tasks, no reliable transfer to vocoded speech perception was found. Trained listeners showed a slight improvement on vocoded consonant identification, yet this did not substantially exceed the (non-significant) learning observed in the untrained controls. Furthermore, we failed to find significant correlations between listeners’ initial learning on vocoded VCVs and their performance on AM tasks. It is worth noting that the sample size of this study as well as the studies from which the initial hypothesis are drawn are small. This might lead to inconsistent results across studies given the binary interpretation of the results (significant versus non-significant effects). Here we have reported summary-based descriptive statistics alongside the more traditional inferential statistics as per to show the level of uncertainty associated with our results. As can be observed in the results section (see also S1 Table) confidence intervals from pre- to post-tests are rather wide and overlapping between groups. This thus suggest that although there might be a tendency for improvement on vocoded VCV identification after AM(R)D training, this is not quite consistent across participants and therefore not observable in studies with sample sizes similar to that of this study and studies of previous literature [17, 20, 21, 61].

We specifically chose the vocoded consonant identification task as an outcome measure because, lacking lexico-semantic cues, it presumably relies more heavily on bottom-up, perceptual processing compared with more complex speech materials such as words or sentences. We reasoned that the effects arising from training on TE cues would be strongest for a task that relies on low-level processing. However, the choice of speech material and the experimental procedure may also have hindered our ability to attain observable transfer of learning. Given the closed set of stimuli, the benefits of longer term AM training might be dwarfed by the rapid learning that occurs via repeated exposure to the stimuli (see [19]). This learning can be also viewed as a carry-over effect from the pre-training which is sometimes observed for several days after perceptual training has ceased [64], and would thus affect trained and control groups equally. Alternatively, participants may have received significant training on vocoded VCV during the pre-training session (overall pre-training reception of Manner: 76%, Voice: 72% and Place: 32%), reaching near-ceiling performance and leaving little room for additional improvement due to training on the AM tasks (see Fig 3 and S1 Table). Fu et al (2005) showed no reliable improvement in spectrally shifted vowel perception after 5 days of training on vowel recognition in CVC contexts and on spectrally shifted monosyllabic words. Although in their study they used 8-channel spectrally shifted and unshifted materials instead of tone-vocoded stimuli, it is possible that similar ceiling effects might be occurring in our study. However, we did find a significant improvement during the task as shown by a significant effect of trial order, suggesting that there was still room for improvement. We ran an additional group trained on AMD task during 5 consecutive days to investigate whether performance could improve with more training. Results again failed to show any reliable benefit with increased training (see S3 Appendix).

Although both of these explanations are plausible, a third alternative should be considered: that the mechanisms involved in learning to distinguish of AM tones are not the same as those involved in learning of vocoded VCVs. This interpretation is supported by the lack of correlation between initial thresholds on AM tasks and learning of vocoded VCV during pre-training. Furthermore, correlation analysis between the amount of learning for each trained group and the amount of generalization to VCV perception also fail to show a significant relationship (AMD-trained: r = -.17, p = .54; AMR-trained: r = .13, p = .61). Hence, it could be hypothesized that whilst perception of TE information relies on detection and discrimination of low-level acoustic features, perceptual learning of vocoded speech involves adjusting the mapping of low-level acoustic feature cues present in the degraded speech signal onto pre-existent phonological representations (e.g., [1, 23, 24, 65, 66]). According to this account, it is not the detection/discrimination of the TE cues that is critical for learning to perceive vocoded speech but rather how those cues map onto higher-level representations.

We found no transfer of learning between the AMD and AMRD tasks even though they used very similar stimuli and identical testing paradigms. Maidment et al. [29] also showed that training on AMD with two different modulation rates (similar to our AMD training paradigm) transferred to an AMD task with a different modulation rate but only partially to an AMRD task with the same modulation rate. Furthermore, in the Maidment et al study [29] AMRD training not only did not transfer to AMD task but also led to disrupted learning of a simultaneously trained AMD task. Wright and Fitzgerald [61] also failed to find generalization to AMD and FD tasks after AMRD training. It is perhaps not surprising, therefore, that training on AM tasks did not transfer to a completely different task that required the identification of vocoded VCVs.

A final possibility is that transfer was not achieved because the AM training was limited to modulations frequencies ≤ 16 Hz and a single carrier frequency, whereas the VCV stimuli contained modulation information up to ~45 Hz along several carrier frequencies (or bands). Since AM learning can be both carrier- [25] and modulation-frequency specific [31], training on a broader range of modulation- and carrier-frequencies might be required to observe transfer of learning to vocoded consonant identification.

Our findings cast doubts on the role of low-level perceptual learning in tone-vocoded consonant perception beyond initial and rapid learning. We showed that the ability of naïve listeners to process TE cues was not related to the improvement in their ability to perceive vocoded nonsense syllables, and that although AM training as used here improved perception of VCV speech material, improvement did not substantially differ from that observed for the untrained control group. Hence, improvements did not appear to be directly related to TE information training in our sample of participants, but rather to repeated exposure to the specific speech stimuli (see [19]). We therefore conclude that training on TE cues in our study does not seem to provide consistent and reliable benefits for the perception of sub-lexical vocoded speech. Training regimens with vocoded speech along the linguistic hierarchy could be explored to find the minimum unit required for training (see [22–26, 28, 38, 67]). Alternatively, as other linguistic features, such as rhythm, prosody and segmental cues in continuous speech have previously been linked with TE information [43, 68, 69], it is possible that transfer of learning after training on AM tasks could be observed for more complex or meta-linguistic aspects of vocoded speech.

## Supporting information

S1 Appendix Specification of statistical models and analyses. Detailed information of the statistical models employed, including tables with results and significance values.
(PDF)

S2 Appendix Phonetic features and information transfer (IT) computation. Detailed information of the consonants used in the experiment as a function of manner, voice and place of articulation and the procedure to compute IT scores, including script written in R.
(PDF)

S3 Appendix Statistical analyses: Pilot group with extended AMD training. (PDF)

S1 Dataset Thresholds obtained from psychophysical tasks and digit span scores. (TXT)

S2 Dataset Raw data obtained from the vocoded consonant identification task. Participants’ scores for all blocks of vocoded consonant identification task, including hits and misses for each specific phonetic feature.
(TXT)

S1 Table Vocoded consonant identification. Means, standard deviations, and 95% confidence intervals fore each group and session.
(PDF)

S2 Table Pearson’s correlation coefficients between tasks. Correlations between hearing and cognitive tasks before undertaking any training.
(PDF)

S1 Audio File Example of vocoded VCV stimuli. File containing vocoded /ama/ non-sense syllable.
(WAV)

S2 Audio File Example of vocoded VCV stimuli. File containing vocoded /isi/ non-sense syllable.
(WAV)

S3 Audio File Example of vocoded VCV stimuli. File containing vocoded /ugu/ non-sense syllable.
(WAV)

S4 Audio File Example of clear VCV stimuli. File containing clear /ama/ non-sense syllable.
(WAV)

S5 Audio File Example of clear VCV stimuli. File containing clear /isi/ non-sense syllable.
(WAV)

S6 Audio File Example of clear VCV stimuli. File containing clear /ugu/ non-sense syllable.
(WAV)

S1 Fig Threshold estimates for each psychophysical task. Means, standard deviations, and 95% confidence intervals fore each group and session.
(PDF)
