# Supplementary material for: Does training with amplitude modulated tones affect tone-vocoded speech perception?
Source: PLoS One. 2019 Dec 27;14(12):e0226288. doi: 10.1371/journal.pone.0226288 (PMC6934405; doi:10.1371/journal.pone.0226288)
Supplement: S1 Appendix — Detailed information of the statistical models employed, including tables with results and significance values. (PDF) [file pone.0226288.s001.pdf]

S1 Appendix. Specification of statistical models and analyses

Pre-test to post-test changes in the variables of interest across groups were analyzed using mixed-effects models. All models included maximal order interactions and main effects for all within-unit predictors as random slopes (where applicable), and random intercepts for subject (S), and item (I). Control group and pre-test were used as reference levels for the evaluation of initial models.

A. Model specifications

Model specifications are provided using the formula notation in R syntax.

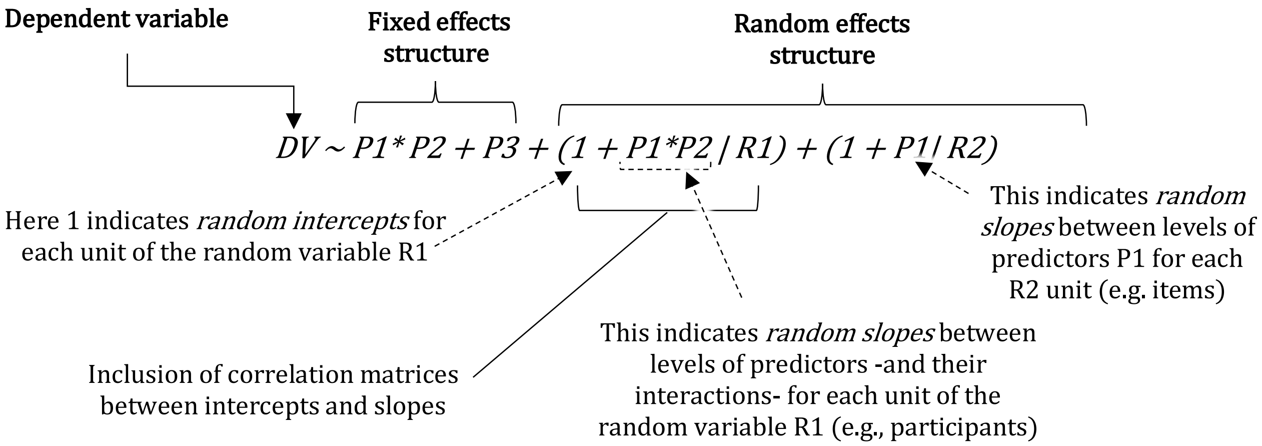

Note that in R syntax the asterisk (*) indicates inclusion of main effects and all interaction terms (either for fixed effects or for the random structure), whilst colon (:) indicates only the inclusion of the higher order interaction term. For instance:

$$DV \sim P1 * P2 + P3 + (1 + P1 * P2 \mid S)$$

Is the same than:

$$DV \sim P1 + P2 + P1{:}P2 + P3 + (1 + P1 + P2 + P1{:}P2 \mid S)$$

Psychophysical tasks

Thresholds (*Y*) for each task were fitted with linear mixed-effects models as follows:

$$Y \sim G * T + C + (1 + T \,|\, S)$$

Where *G* (here and thereafter) is the predictor group (control, AMR-trained, AMD-trained), T is the predictor test (pre-test, post-test), and *C* is the control predictor block (block 1, block 2). In the presence of significant group × test interaction, models were refitted with the differences of learning between pre- to post-test. This allowed us to investigate whether the magnitude of learning due to training was significantly different across groups. We thus compute the learning effect as follows:

$$Y \sim G + C + (1 \,|\, S)$$

Where *Y* here represents the magnitude of learning. In order to account for the variability in learning between tests and between blocks, the control variable block was also included.

Vocoded consonant identification

Responses (*Y*) on consonants (identification accuracy) were fitted with mixed-effects logit model as follows:

$$Y \sim G * T + C + (1 + T \,|\, S) + (1 + G * T \,|\, I)$$

Where C represents the control continuous variable trial-order (i.e., order of presentation for each subject of each 60 vocoded VCVs).

Relative information transfer percentages

Percentages obtained from relative Information transfer analyses (*Y*) were fitted with linear mixed-effects models as follows:

$$Y \sim G * T * P + (1 + T \,|\, S)$$

Where *P* corresponds to the predictor type of phonetic feature (voice, manner, place). Note that given the number of data points obtained from relative IT analysis, the maximal within-unit random slopes model was impracticable and thus, only the within-unit predictor T (Test) was include in the random structure of the model.

B. Statistical analyses results

For each task we first present the summary of the results from the initial model (including fixed and random effects), followed by the analysis of variance table, and the differences of the least squared means table for the conditions of interest. Magnitude of learning effects results are then presented whenever there was a significant interaction between the predictors group and pre- to post-test.

Amplitude modulation-depth discrimination (AMD) outputs

Table A. AMD task outputs. Linear mixed model fit by REML. T-tests use Satterthwaite approximations to degrees of freedom

Random effects:

Groups	Name	Variance	Std.Dev.
**Subject**	**(Intercept)**	0.05422	0.2329
	**Testpost**	0.0177	0.1331
	**Residual**	0.01566	0.1251

Number of observations: 205, groups: Subject, 53

Fixed effects:

	Estimate	Std. Error	DF	t value	Pr(>|t|)	
**(Intercept)**	0.938728	0.059371	51.55	15.811	<2e-16	***
**GroupAMD-trained**	0.0659	0.084515	49.89	0.78	0.4392	
**GroupAMR-trained**	0.008966	0.083273	49.83	0.108	0.9147	
**Testpost**	-0.0796	0.043237	48.95	-1.841	0.0717	.
**Block2**	0.003877	0.017628	101.42	0.22	0.8264	
**GroupAMD:Testpost**	-0.13467	0.062418	49.97	-2.158	0.0358	*
**GroupAMR:Testpost**	0.005542	0.061476	49.85	0.09	0.9285	

*Significance codes: *** (p<.001), ** (p<.01), * (p<0.05), · (p<.1)*

Table B. Analysis of Variance Table of type III for AMD task. Satterthwaite approximation for degrees of freedom.

	Sum Sq	Mean Sq	NumDF	DenDF	F.value	Pr(>F)	
**Group**	0.0008	0.0004	2	50.227	0.0256	0.97471	
**Test**	0.36514	0.36514	1	50.219	23.3184	1.33E-05	***
**Block**	0.00076	0.00076	1	101.424	0.0484	0.82637	
**Group:Test**	0.09963	0.04982	2	50.214	3.1814	0.05002	.

*Significance codes: *** (p<.001), ** (p<.01), * (p<0.05), · (p<.1)*

Table C. Differences of Least Squared Means in AMD task. Learning effect for each training group.

Group	Test	Estimate	Std. Error	DF	t-value	Lower CI	Upper CI	p-value	
**Control**	**pre-post**	0.0796	0.0432	49	1.84	-0.0073	0.1665	0.0717	.
**AMD-trained**	**pre-post**	0.2143	0.045	50.9	4.76	0.1239	0.3046	<2e-16	***
**AMR-trained**	**pre-post**	0.0741	0.0437	50.8	1.69	-0.0137	0.1618	0.0963	.

*Significance codes: *** (p<.001), ** (p<.01), * (p<0.05), · (p<.1)*

Magnitude of learning effects across groups

Table D. Magnitude of learning effects in AMD task. Linear mixed model fit by REML. T-tests use Satterthwaite approximations to degrees of freedom.

Random effects:

Groups	Name	Variance	Std.Dev.
**Subject**	**(Intercept)**	0.0194	0.1393
	**Residual**	0.02774	0.1666

Number of obs: 99, groups: Subject, 53

Fixed effects:

	Estimate	Std. Error	DF	t value	Pr(>|t|)	
**(Intercept)**	0.09067	0.04682	63.34	1.937	0.0572	.
**GroupAMD-trained**	0.13003	0.06281	49.98	2.07	0.0436	*
**GroupAMR-trained**	-0.01214	0.06182	49.8	-0.196	0.8451	
**Block2**	-0.02648	0.03403	49.23	-0.778	0.4401	

*Significance codes: *** (p<.001), ** (p<.01), * (p<0.05), · (p<.1)*

Table E. Magnitude of the effect in AMD task. Differences of Least Squared Means between groups.

Groups		Estimate	Std. Error	DF	t-value	Lower CI	Upper CI	p-value	
**Control**	**- AMD-trained**	-0.13	0.0628	50	-2.07	-0.2562	-0.0039	0.0436	*
**Control**	**- AMR-trained**	0.0121	0.0618	49.8	0.2	-0.112	0.1363	0.8451	
**AMD-trained**	**- AMR-trained**	0.1422	0.0633	51.2	2.25	0.0151	0.2693	0.0291	*

*Significance codes: *** (p<.001), ** (p<.01), * (p<0.05), · (p<.1)*

Amplitude modulation-rate discrimination (AMR) outputs

Table F. AMR task outputs. Linear mixed model fit by REML**.** T-tests use Satterthwaite approximations to degrees of freedom.

Random effects:

**Groups**	**Name**	**Variance**	**Std.Dev.**
**Subject**	**(Intercept)**	0.02826	0.1681
	**Testpost**	0.01025	0.1013
	**Residual**	0.01517	0.1231

Number of obs: 206, groups: Subject, 53

Fixed effects:

	**Estimate**	**Std.Error**	**DF**	**t value**	**Pr(>|t|)**	
**(Intercept)**	1.35434	0.04572	53.01	29.623	< 2e-16	***
**GroupAMD-trained**	-0.0572	0.06433	49.08	-0.889	0.37824	
**GroupAMR-trained**	-0.03437	0.06354	49.44	-0.541	0.591	
**Testpost**	-0.1047	0.03782	48.93	-2.769	0.00793	**
**Block2**	-0.07022	0.01728	98.93	-4.064	9.69e-05	***
**GroupAMD:Testpost**	0.01295	0.05444	49.48	0.238	0.81304	
**GroupAMR:Testpost**	-0.12969	0.05381	49.94	-2.41	0.01968	*

*Significance codes: *** (p<.001), ** (p<.01), * (p<0.05), · (p<.1)*

Table G. Analysis of Variance Table of type III in AMR task. Satterthwaite approximation for degrees of freedom.

	**Sum Sq**	**Mean Sq**	**NumDF**	**DenDF**	**F.value**	**Pr(>F)**	
**Group**	0.05282	0.02641	2	49.28	1.742	0.18585	
**Test**	0.63551	0.63551	1	49.957	41.905	4.01E-08	***
**Block**	0.25045	0.25045	1	98.934	16.515	9.686E-05	***
**Group:Test**	0.1279	0.06395	2	49.956	4.217	0.02032	*

*Significance codes: *** (p<.001), ** (p<.01), * (p<0.05), · (p<.1)*

Table H. Differences of Least Squared Means in AMR task. Learning effect for each training group.

**Group**	**Test**	**Estimate**	**Std. Error**	**DF**	**t-value**	**Lower CI**	**Upper CI**	**p-value**
**Control**	**pre-post**	0.1047	0.0378	48.9	2.77	0.0287	0.1807	0.0079 **
**AMD-trained**	**pre-post**	0.0918	0.0392	50	2.34	0.0131	0.1704	0.0232 *
**AMR-trained**	**pre-post**	0.2	0.0383	51	6.12	0.1575	0.311	<2e-16 ***

*Significance codes: *** (p<.001), ** (p<.01), * (p<0.05), · (p<.1)*

Magnitude of learning effects across groups

Table I. Magnitude of learning effects in AMR task. Linear mixed model fit by REML. t-tests use Satterthwaite approximations to degrees of freedom

Random effects:

Groups	Name	Variance	Std.Dev.
**Subject**	**(Intercept)**	0.01053	0.1026
	**Residual**	0.03005	0.1734

Number of obs: 100, groups: Subject, 53

Fixed effects:

	Estimate	Std. Error	DF	t value	Pr(>|t|)	
**(Intercept)**	0.10529	0.042161	67.76	2.497	0.0149	*
**GroupAMD-trained**	-0.031544	0.055001	48.94	-0.574	0.5689	
**GroupAMR-trained**	0.12633	0.05443	49.55	2.321	0.0244	*
**Block2**	0.002976	0.034976	49.93	0.085	0.9325	

*Significance codes: *** (p<.001), ** (p<.01), * (p<0.05), · (p<.1)*

Table J. Magnitude of the effects in AMR task. Differences of Least Squared Means between groups.

Groups		Estimate	Std. Error	DF	t-value	Lower CI	Upper CI	p-value	
**Control**	**- AMD-trained**	0.0315	0.055	48.9	0.57	-0.079	0.1421	0.5689	
**Control**	**- AMR-trained**	-0.1263	0.0544	49.5	-2.32	-0.2357	-0.017	0.0244	*
**AMD-trained**	**- AMR-trained**	-0.1579	0.0556	50.3	-2.84	-0.2695	-0.0463	0.0065	**

*Significance codes: *** (p<.001), ** (p<.01), * (p<0.05), · (p<.1)*

Frequency discrimination (FD) outputs

Table K. FD task outputs. Linear mixed model fit by REML. T-tests use Satterthwaite approximations to degrees of freedom.

Random effects:

Groups	Name	Variance	Std. Dev.
**Subject**	**(Intercept)**	0.12344	0.35134
	**Testpost**	0.00474	0.06885
	**Residual**	0.03451	0.18578

Number of obs: 209, groups: Subject, 53

Fixed effects:

	Estimate	Std.Error	DF	t value	Pr(>|t|)	
**(Intercept)**	0.233105	0.089345	52.38	2.609	0.011807	*
**GroupAMD-trained**	0.007178	0.125031	50.28	0.057	0.954446	
**GroupAMR-trained**	-0.158317	0.126856	50.28	-1.248	0.21781	
**Testpost**	-0.176439	0.047102	103.45	-3.746	0.000296	***
**Block2**	-0.019471	0.025777	152.22	-0.755	0.451212	
**GroupAMD:Testpost**	0.099956	0.066608	103.44	1.501	0.136493	
**GroupAMR:Testpost**	0.102203	0.067598	103.48	1.512	0.133604	

*Significance codes: *** (p<.001), ** (p<.01), * (p<0.05), · (p<.1)*

Table L. Analysis of Variance Table of type III in FD task. Satterthwaite approximation for degrees of freedom.

	Sum Sq	Mean Sq	NumDF	DenDF	F.value	Pr(>F)	
**Group**	0.05306	0.02653	2	50.082	0.7687	0.4689902	
**Test**	0.54403	0.54403	1	103.483	15.7628	0.0001328	***
**Block**	0.01969	0.01969	1	152.224	0.5705	0.4512125	
**Group:Test**	0.10493	0.05246	2	103.467	1.5201	0.2235392	

*Significance codes: *** (p<.001), ** (p<.01), * (p<0.05), · (p<.1)*

Table M. Differences of Least Squared Means in FD task. Learning effect for each training group.

Group	Test	Estimate	Std. Error	DF	t-value	Lower CI	Upper CI	p-value	
**Control**	**pre-post**	0.1764	0.0471	103.4	3.75	0.083	0.2698	0.0003	***
**AMD-trained**	**pre-post**	0.0765	0.0471	103.4	1.62	-0.0169	0.1699	0.1075	
**AMR-trained**	**pre-post**	0.0742	0.0485	103.5	1.53	-0.0219	0.1704	0.1288	

*Significance codes: *** (p<.001), ** (p<.01), * (p<0.05), · (p<.1)*

Vocoded consonant identification outputs

Table N. Consonant identification outputs. Generalized linear mixed model fit by maximum likelihood (Laplace Approximation).

Random effects:

Groups	Name	Variance	Std. Dev.
**VCV**	**(Intercept)**	1.48046	1.21674
	**GroupAMD-trained**	0.055288	0.23513
	**GroupAMR-trained**	0.341306	0.58421
	**Testpost**	0.034228	0.18501
	**GroupAMD:Testpost**	0.03308	0.18188
	**GroupAMR:Testpost**	0.025515	0.15973
**Subject**	**(Intercept)**	0.737514	0.85879
	**Testpost**	0.009307	0.09647

Number of obs: 6120, groups: VCV, 60; Subject, 51

Fixed effects:

	Estimate	Std. Error	z value	Pr(>|t|)	
**(Intercept)**	0.076599	0.273497	0.28	0.779423	
**GroupAMD-trained**	0.298943	0.316573	0.944	0.34501	
**GroupAMR-trained**	0.488332	0.321045	1.521	0.128243	
**Testpost**	0.175022	0.113781	1.538	0.12399	
**Trial**	0.00682	0.001928	3.538	0.000403	***
**GroupAMD:Testpost**	0.140304	0.166013	0.845	0.398033	
**GroupAMR:Testpost**	0.188176	0.16884	1.115	0.265056	

*Significance codes: *** (p<.001), ** (p<.01), * (p<0.05), · (p<.1)*

Table O. Analysis of Deviance Table, Type III Wald χ^2 tests, in vocoded consonant identification task. Since conditional Wald χ^2 main effects are biased towards the reference level used in the interaction term, results from each group used as reference are reported.

Reference	Control			AMR-trained			AMD-trained		
	χ^2	DF	Pr(>χ^2)	χ^2	DF	Pr(>χ^2)	χ^2	DF	Pr(>χ^2)
**(Intercept)**	0.0784	1	0.779423	3.1644	1	0.075261	1.5926	1	0.2069493
**Group**	2.386	2	0.3033095	2.3842	2	0.303577	2.3843	2	0.3035695
**Test**	2.3662	1	0.12399	8.2552	1	0.004064	6.4832	1	0.0108896
**Trial**	12.5159	1	0.0004035	12.5162	1	0.000403	12.516	1	0.0004035
**Group:Test**	1.3911	2	0.4988052	1.3914	2	0.498714	1.3913	2	0.4987603

Table P. Differences of Least Squared Means in vocoded consonant identification task.

Group	Test	Estimate	Std Error	z. ratio	p-value	
**Control**	**pre-post**	0.1750221	0.1137808	1.53824	0.124	
**AMD-trained**	**pre-post**	0.3153265	0.1238417	2.546207	0.0109	*
**AMR-trained**	**pre-post**	0.3631977	0.1264144	2.873071	0.0041	**

*Significance codes: *** (p<.001), ** (p<.01), * (p<0.05), · (p<.1)*

Information transfer analysis outputs

Table Q. Relative IT outputs. Linear mixed model fit by REML. T-tests use Satterthwaite approximations to degrees of freedom

Random effects:

Groups	Name	Variance	Std.Dev.
**Subject**	**(Intercept)**	346.847	18.624
	**Testpost**	6.334	2.517
	**Residual**	132.191	11.497

Number of obs: 306, groups: Subject, 51

Fixed effects:

	Estimate	Std.Error	DF	t value	Pr(>|t|)	
**(Intercept)**	71.9056	5.1588	71.32	13.938	<2e-16	***
**GroupAMD-trained**	8.484	7.5202	71.32	1.128	0.263	
**GroupAMR-trained**	10.7718	7.4022	71.32	1.455	0.15	
**Testpost**	3.7565	3.8781	232.3	0.969	0.334	
**TypePlace**	-42.9642	3.8325	240	-11.211	<2e-16	***
**TypeVoice**	-2.1618	3.8325	240	-0.564	0.573	
**GroupAMD:Testpost**	-0.7657	5.6533	232.3	-0.135	0.892	
**GroupAMR:Testpost**	1.213	5.5645	232.3	0.218	0.828	
**GroupAMD:TypePlace**	-3.4716	5.5867	240	-0.621	0.535	
**GroupAMR:TypePlace**	-6.3137	5.4991	240	-1.148	0.252	
**GroupAMR:TypeVoice**	-6.8185	5.5867	240	-1.22	0.223	
**GroupAMR:TypeVoice**	-4.3539	5.4991	240	-0.792	0.429	
**Testpost:TypePlace**	-0.3651	5.4199	240	-0.067	0.946	
**Testpost:TypeVoice**	3.1254	5.4199	240	0.577	0.565	
**GroupAMD:Testpost:TypePlace**	4.0581	7.9008	240	0.514	0.608	
**GroupAMR:Testpost:TypePlace**	1.4696	7.7769	240	0.189	0.85	
**GroupAMD:Testpost:TypeVoice**	5.1169	7.9008	240	0.648	0.518	
**GroupAMR:Testpost:TypeVoice**	-0.5679	7.7769	240	-0.073	0.942	

*Significance codes: *** (p<.001), ** (p<.01), * (p<0.05), · (p<.1)*

Table L. Analysis of Variance Table of type III for relative IT. Satterthwaite approximation for degrees of freedom.

	Sum Sq	Mean Sq	NumDF	DenDF	F. value	Pr(>F)	
**Group**	251	126	2	48.001	0.95	0.3937	
**Test**	2517	2517	1	155.771	19.04	< 2.3e-05	***
**Type**	130234	65117	2	239.997	492.6	< 2.2e-16	***
**Group:Test**	65	33	2	155.771	0.25	0.7814	
**Group:Type**	432	108	4	239.997	0.82	0.5158	
**Test:Type**	286	143	2	239.997	1.08	0.3405	
**Group:Test:Type**	91	23	4	239.997	0.17	0.9525	

*Significance codes: *** (p<.001), ** (p<.01), * (p<0.05), · (p<.1)*
