# Supplementary material for: Does training with amplitude modulated tones affect tone-vocoded speech perception?
Source: PLoS One. 2019 Dec 27;14(12):e0226288. doi: 10.1371/journal.pone.0226288 (PMC6934405; doi:10.1371/journal.pone.0226288)
Supplement: S2 Appendix — Detailed information of the consonants used in the experiment as a function of manner, voice and place of articulation and the procedure to compute IT scores, including script written in R. (PDF) [file pone.0226288.s002.pdf]

S2 Appendix. Phonetic features and information transfer (IT) computation

A. Phonetic features

Orthographic and phonological representations for each consonant used in the experiment are given in Table A. Each consonant was classified according to three different phonetic features: Voice, Manner and Place (see Table B) and individual confusions matrices were computed accordingly.

Table A. Orthographic and phonological representations for each consonant used in the experiment.

**Consonants**	B	CH	D	F	G	J	K	L	M	N	P	R	S	SH	T	TH	V	W	Y	Z
**Sound correspondence**	/b/	/ʧ/	/d/	/f/	/g/	/ʤ/	/k/	/l/	/m/	/n/	/p/	/r/	/s/	/ʃ/	/t/	/θ/	/v/	/w/	/j/	/z/

Table B. Distribution of categories across phonetic features

**Manner**	**Voice**	**Place of articulation**		
		**Front**	**Middle**	**Back**
**Plosives***	**Voiced**	/b/	/d/	/g/ /ʧ/
	**Voiceless**	/p/	/t/	/k/ /ʤ/
**Fricatives**	**Voiced**	/v/	/z/	
	**Voiceless**	/f/ /θ/	/s/	/ʃ/
**Nasals**	**Voiced**	/m/	/n/	
**Approximants**	**Voiced**	/w/	/l/ /r/	/j/

* *Consonants starting with a plosive sound*

B. Relative information transfer (IT).

Relative IT was computed from individual confusion matrices for each phonetic feature and session (see raw data in S2 Dataset and R script in section C, below). IT was defined by:

$$\mathrm{IT}(s;r) = MLP(s) + MLP(r) - MLP(sr)$$
$$= \sum_{s} p_s \log_2 p_s + \sum_{r} p_r \log_2 p_r - \sum_{s,r} p_{sr} \log_2 p_{sr} \tag{1}$$

Where *s* corresponds to the input, *r* to the output, and MLP to the mean logarithmic probabilities. Since the true probabilities (p) are not known they were estimated from the relative frequencies obtained during the experiment. Therefore, the maximum likelihood estimate of $\mathrm{IT}(s;r)$ is obtained by dividing the number of times a given event was observed (n) by the total number of observations (N). Thus, p_r can be defined as n_r (i.e., the number of times output *r* was present) divided by *N*, and similarly for p_s and p_{sr}.

Relative IT was defined as the ratio of transmitted information, $\mathrm{IT}(s;r)$, to the input entropy, H_s

$$IT_{rel}(s;r) = \frac{IT(s;r)}{H_s} \tag{2}$$

Where H_s reflects the amount of uncertainty associated with the value of *s*. Relative IT thus can be defined as

$$IT_{rel}(s;r) = \frac{-\sum_s \frac{n_s}{N} \log_2 \frac{n_s}{N} - \sum_r \frac{n_r}{N} \log_2 \frac{n_r}{N} + \sum_s \sum_r \frac{n_{sr}}{N} \log_2 \frac{n_{sr}}{N}}{-\sum_s p_s \log p_s} \tag{3}$$

Where p_s reflects the probability of each category to occur (within a given phonetic feature) given the number of instances of each consonant in each category.

In confusion matrices where *r* was 0 (e.g., the listener did not receive any bit of information available for a given level of a specific phonetic feature) the expression of the form $p \log_2 p$ was considered = 0. This is justified because for any logarithmic base:

$$\lim_{p \to 0+} p \log_2 p = 0$$

C. R script to calculate relative IT from individual confusion matrices

```
########################################################################################
# Step 1: quantify the relative transmission of information for each subject in each experimental session
########################################################################################
#see README file for specifications of the data.frame.

# call function (see function below - Step 0) //
source("~/IT.fnc.txt")

#set common parameters across analyses
n=60 #total number of trials (S-R combinations)

############# Rel.IT for Voice ###########
#set parameters for the specific phonetic feature
le=levels(D$Voice.x) # levels of the phonetic feature
list_IT=list()

#set table with instances for each consonant as a function of levels of the factor of interest.
P=table(D[D$Session==4,]$Voice.x, D[D$Session==4,]$C) # Select only one of the sessions

# Matrix with all percentages of rel. IT for each participant and session
for (j in levels(D$Session)){
  IT_=list()
  for(i in levels(D$SUBJECT)){
    M=table((D[D$Session==j & D$SUBJECT==i,]$Voice.y), (D[D$Session==j & D$SUBJECT==i,]$Voice.x))
# compute confusion matrix
    IT_[[i]]=IT.fnc(M)*100 # call function IT and return % of rel.IT
  }
  list_IT[[j]]=stack(IT_)
}
IT_session=as.data.frame(list_IT)
cn(IT_session)
IT_session=subset(IT_session, select= c(2,1,3,5,7,9))
names(IT_session)=c('SUBJECT', 'S2_V','S3_V','S4_V', 'S5_V','S6_V')

# Final Voice data with % rel.IT
IT_V=IT_session


############# Rel. IT for Manner ###########
#set paramaters
le=levels(D$Manner.x) # levels of Stimuli
list_IT=list()

#set table with instances for each consonant as a function of levels of the factor of interest
P=table(D[D$Session==4,]$Manner.x, D[D$Session==4,]$C)

# Matrix with all percentage of relative IT for each participant and session
for (j in levels(D$Session)){
  IT_=list()
  for(i in levels(D$SUBJECT)){
    M=table((D[D$Session==j & D$SUBJECT==i,]$Manner.y), (D[D$Session==j &
D$SUBJECT==i,]$Manner.x)) # create individual confusion matrices
    IT_[[i]]=IT.fnc(M)*100 # call function IT and return %
  }
  list_IT[[j]]=stack(IT_)
}
```

```
IT_session=as.data.frame(list_IT)
cn(IT_session)
IT_session=subset(IT_session, select= c(2,1,3,5,7,9))
names(IT_session)=c('SUBJECT', 'S2_M','S3_M','S4_M', 'S5_M','S6_M')

# Final Manner data with % IT
IT_M=IT_session

############# Rel. IT for Place  ###########
#set paramaters
le=levels(D$Place.x) # levels of phonetic feature
list_IT=list()

#set table with instances for each consonant as a function of levels of the factor of interest
P=table(D[D$Session==4,]$Place.x,D[D$Session==4,]$C)

# Matrix with all percentage of relative IT for each participant and session.
for (j in levels(D$Session)){
  IT_=list()
  for(i in levels(D$SUBJECT)){
    M=table((D[D$Session==j & D$SUBJECT==i,]$Place.y), (D[D$Session==j & D$SUBJECT==i,]$Place.x))
# create individual confusion matrices
    IT_[[i]]=IT.fnc(M)*100 # call function IT and return %
  }
  list_IT[[j]]=stack(IT_)
}
IT_session=as.data.frame(list_IT)
cn(IT_session)
IT_session=subset(IT_session, select= c(2,1,3,5,7,9))
names(IT_session)=c('SUBJECT', 'S2_P','S3_P','S4_P', 'S5_P','S6_P')

# Final matrix for Place = % rel. IT
IT_P=IT_session

#end

########################################################################################
#Step 0 Function that computes IT and relative IT scores from individual matrices.
################################### IT.fnc ############################################

IT.fnc=function(x){
  Sb=vector()
  Rb=vector()
  SRd=vector()
  Px=vector()

  #function to solve the 0*(-Inf) solution
  logNAN.fnc=function(y){
    L2=log2(y)
    if(L2=='-Inf'){
      L2=0
      warning (paste('In',i,'|Session',j,'Log2(0)=-Inf;-Inf*0==0 into LME(s,r) equation')) # where i=subject and
j=session
    }

    return(L2)
  }

  for (b in le){ # b is levels in confusion matrix (‘le’ has to be defined by user)
    #MLE - input
```

```
   Sb[b]=sum(sum(M[,b])/n*log2(sum(M[,b])/n)) # MLE(S(b))  S refers to Stimulus (TP + FN, sum of each columns)
   #MLE - output
   Rb[b]=sum(M[b,])/n*logNAN.fnc(sum(M[b,])/n) # MLE(R(b)) where R refers to Response in confusion matrix (TP + FP, sum of each row)

   #probability of each level to occur each 20 distinctive consonants
   Px[b]= sum(P[b,])/sum(P)*log2(sum(P[b,])/sum(P)) # P is a table defined by user that represents the probability of each level to occur within the 20 different consonants.
  }

  #MLE - joint (input|output)
  for (d in le){
   SRg=vector()
   for(g in le){
     SRg[g]=sum(M[d,g]/n*logNAN.fnc(M[d,g]/n))# # MLE(S==1rst level: R==all levels)) where SRg is S - R intersection (cell) in confusion matrix

     SRG=sum(SRg)
   }
   SRd[d]=SRG
  }

  # for rel.IT= -(S)-(R)+(SR)/-Hx

  S=sum(Sb) ## MLE(S) -   I Stimuli (Is) defined as input entropy  - maxim likelihood estimate (MLE)
  R=sum(Rb)## MLE(R) -   I Response (Ir) defined as output entropy
  SR=sum(SRd)## MLE(SR) I(x,y) Information S-R defined as joint entropy
  Hx=-(sum(Px)) # the amount of uncertainty associated with X ~ Entropy of S (note the negative). Notice that when using one subject*factor of two levels this is the same than S

  It=-(S)-(R)+(SR) # ~ T(s,r)= MLE(s)+MLE(r)-MLE(sr)
  IT=It/Hx # Relative Information Transfer (rel. IT)

  return(IT)

  return(warning) #
}

#end
############################################################################################
```
