# Supplementary material for: Does training with amplitude modulated tones affect tone-vocoded speech perception?
Source: PLoS One. 2019 Dec 27;14(12):e0226288. doi: 10.1371/journal.pone.0226288 (PMC6934405; doi:10.1371/journal.pone.0226288)
Supplement: S3 Appendix — (PDF) [file pone.0226288.s003.pdf]

S3 Appendix. Statistical analyses: pilot group with extended AMD training (AMD2-trained).

A pilot experiment was run to confirm whether extended training on temporal-envelope cues could potentially transfer to vocoded speech identification. Training was extended to 5 days and participants performed a total of 42 blocks of training on AMD task (60 trials per block) using the same stimuli and materials than in experiment 1. However, after 12 participants performing extended AMD training (AMD2-trained group) the study was aborted due to the lack of reliable and significant benefit. Preliminary results were similar to those reported with shorter training.

Statistical analyses were conducted following the same procedure explained in S1_Appendix. One participant had thresholds deviating more than 2.5 interquartile-range above and below the 3rd and 1st quartile and thus he/she was removed from analyses.

Psychophysical tasks. As expected AMD2-trained group improved on AMD task (β = .20, SE = .05, t = 3.18, p = .002) and AMR task (β = 0.2, SE =.05, t = 3.93, p <.001), but not on FD task (β = .01, SE= .05, t = 1.72, p = .09).

VCV task. There was a trend for improvement in VCV task after training although not significant (β = .24, SE = .14, z = 1.79, p = .07) and this was not significantly different than the other groups (group x test: χ^2 (3) = 1.46, p = .69).

•
