# Supplementary material for: Does training with amplitude modulated tones affect tone-vocoded speech perception?
Source: PLoS One. 2019 Dec 27;14(12):e0226288. doi: 10.1371/journal.pone.0226288 (PMC6934405; doi:10.1371/journal.pone.0226288)
Supplement: S1 Table — Means, standard deviations, and 95% confidence intervals fore each group and session. (PDF) [file pone.0226288.s006.pdf]

S1 Table. Vocoded consonant identification. Means, standard deviations, and 95% confidence intervals fore each group and session.

Session	Group	Mean	SD	95 % CI - Lower	95 % CI - Upper
Pre-training1	Control	36.76	48.24	14.47	59.04
	AMD-trained	38.96	48.79	15.05	62.87
	AMRD-trained	46.46	49.90	22.74	70.18
Pre-training2	Control	55.09	49.76	32.10	78.08
	AMD-trained	58.96	49.22	34.84	83.07
	AMRD-trained	63.33	48.21	40.41	86.25
Pre-test	Control	56.02	49.66	33.08	78.96
	AMD-trained	60.83	48.84	36.90	84.76
	AMRD-trained	63.04	48.29	40.08	86.00
Post-test	Control	58.52	49.29	35.75	81.29
	AMD-trained	65.21	47.66	41.86	88.56
	AMRD-trained	67.75	46.77	45.51	89.98
