# Supplementary material for: Does training with amplitude modulated tones affect tone-vocoded speech perception?
Source: PLoS One. 2019 Dec 27;14(12):e0226288. doi: 10.1371/journal.pone.0226288 (PMC6934405; doi:10.1371/journal.pone.0226288)
Supplement: S2 Table — Correlations between hearing and cognitive tasks before undertaking any training. (PDF) [file pone.0226288.s007.pdf]

S2 Table. Pearson's correlation coefficients between tasks.

Correlations between experimental and cognitive tasks before undertaking any training.

	AMD	AMR	FD	Forward digit span	Backward digit span
**Vocoded VCV learning**	.039	.004	-.031	.008	.099
**AMD**	-	.465***	.525***	-.335*	-.318*
**AMR**	-	-	.425**	-.335*	-.321*
**FD**	-	-	-	-.381**	-.427**
**Forward digit span**	-	-	-	-	.664***
**Initial vocoded VCV performance**	-.147	-.117	-.347*	.354**	.294*

*Vocoded VCV learning was measured by subtracting the percentage of correct consonant identifications in the first block from the second block in Day 1. Initial vocoded VCV performance refers to performance on first block only. For AMD, AMR and FD tasks, only pre-test thresholds were used. Uncorrected significance p-values (two-tailed): ***(p<.001), **(p<.01), *(p<.05)*

The link observed by Erb et al. [1] between the discrimination of AMR and the improvement on the perception of vocoded sentences disappeared when forward digit span was partialled out, suggesting that performance on vocoded sentences recognition was related to the common variance explained by AMR and forward digit span. In our study, although a moderate yet significant correlation was found between AM tasks and digit span tasks (see table above), no correlation was observed between digit span tasks and vocoded VCV learning, or between AM tasks and vocoded consonant identification task.

References

1 Erb J, Henry MJ, Eisner F, Obleser J. Auditory skills and brain morphology predict individual differences in adaptation to degraded speech. Neuropsychologia. 2012;50(9):2154-64.
