# Supplementary material for: Does training with amplitude modulated tones affect tone-vocoded speech perception?
Source: PLoS One. 2019 Dec 27;14(12):e0226288. doi: 10.1371/journal.pone.0226288 (PMC6934405; doi:10.1371/journal.pone.0226288)
Supplement: S1 Fig — Means, standard deviations, and 95% confidence intervals fore each group and session. (PDF) [file pone.0226288.s014.pdf]

S1 Figure. Thresholds estimates for each psychophysical task.

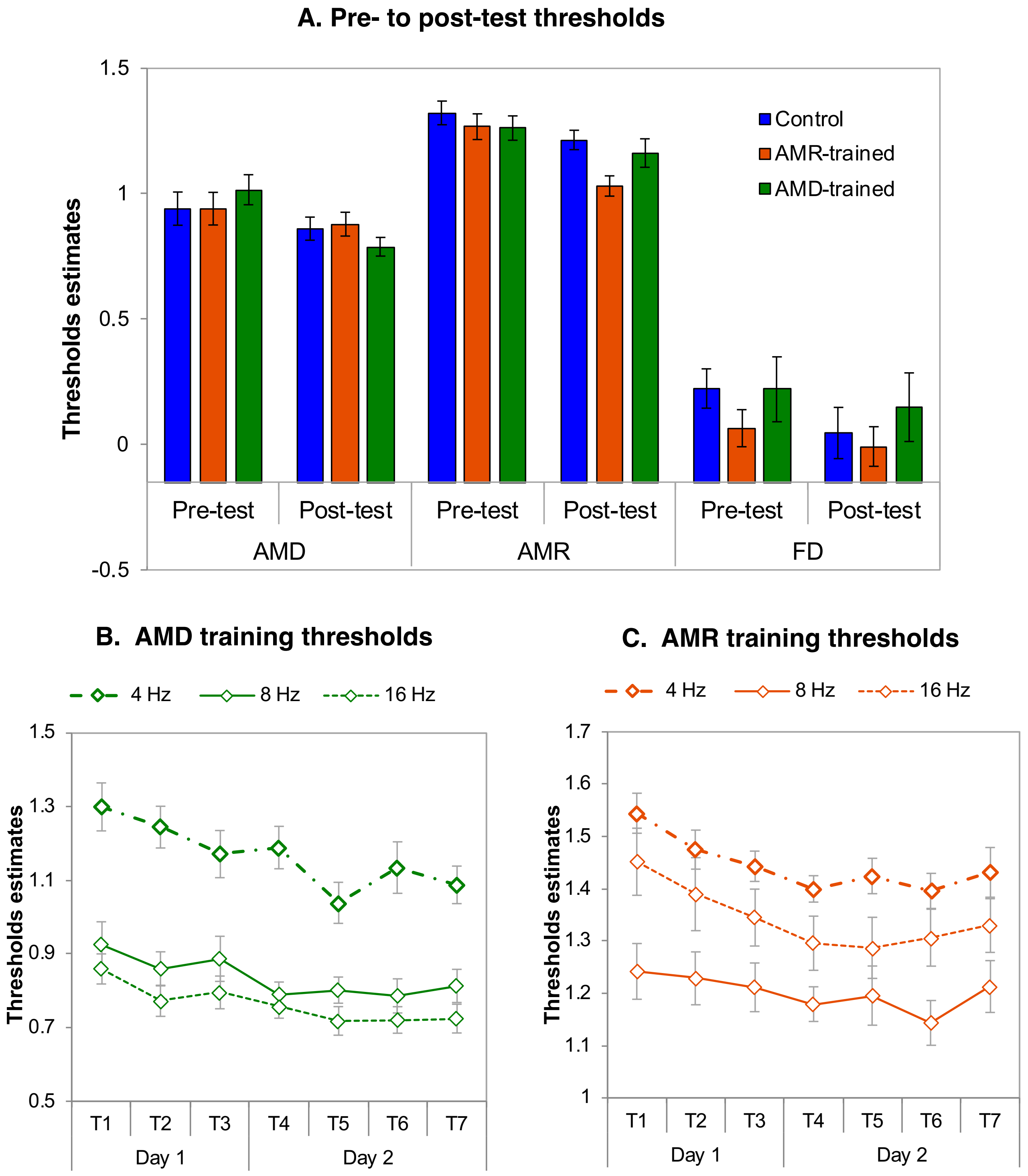

Figure S1. Psychophysical tasks. (A) Mean thresholds estimates for each psychophysical task before and after training for each group of participants. **(B)** Learning trajectory for AMD-trained group across the different modulation rates during training. **(C)** Learning trajectory for AMR-trained group across the different modulation rates during training. Error bars represent +/- 1 standard error of the mean (s.e.m).

Thresholds estimates for each psychophysical task. Means, standard deviations, and 95% confidence interval for each group and session.

	Session	Group	Mean	SD	95 % CI - Lower	95 % CI - Upper
Pre-Training	AMD task	Control	0.94	0.28	0.81	1.07
		AMR-trained	0.94	0.27	0.81	1.07
		AMD-trained	1.02	0.25	0.90	1.13
	AMRD task	Control	1.32	0.20	1.23	1.41
		AMR-trained	1.27	0.20	1.17	1.36
		AMD-trained	1.26	0.15	1.19	1.33
	FD task	Control	0.22	0.20	0.13	0.32
		AMR-trained	0.07	0.21	-0.04	0.17
		AMD-trained	0.22	0.20	0.13	0.32
Post- training	AMD task	Control	0.86	0.24	0.75	0.97
		AMR-trained	0.88	0.23	0.77	0.99
		AMD-trained	0.79	0.22	0.68	0.89
	AMRD task	Control	1.21	0.31	1.07	1.36
		AMR-trained	1.03	0.29	0.89	1.17
		AMD-trained	1.16	0.31	1.02	1.31
	FD task	Control	0.05	0.18	-0.04	0.13
		AMR-trained	-0.01	0.21	-0.11	0.09
		AMD-trained	0.15	0.23	0.04	0.26
